# Implications of pentose phosphate metabolism and astrocyte co-expression patterns in the pathogenesis of Alzheimer’s disease: evidence from artificial intelligence-driven omics and clinical validation

**DOI:** 10.3389/fnins.2026.1887060

**Published:** 2026-09-08

**Authors:** Chao Gong

**Affiliations:** School of Life Sciences, Yangtze University, Jingzhou, Hubei, China

**Keywords:** Alzheimer’s disease, artificial intelligence, astrocytes, multi-omics, pentose phosphate metabolism

## Abstract

**Background:**

Metabolic reprogramming in glial cells is increasingly recognized as a pivotal factor in the pathogenesis of AD. While both the pentose phosphate pathway (PPP), a crucial metabolic route for redox balance and biosynthesis, and astrocyte reactivity are implicated in AD, their integrated molecular interaction between processes remains largely uncharted.

**Methods:**

We employed an integrated multi-omics and artificial intelligence (AI) framework. First, we applied the Limma, WGCNA, and xCell algorithms to bulk RNA-seq profiles from the hippocampus of AD patients to identify a gene signature linking the PPP and astrocyte reactivity (PA). This signature was then used to construct a diagnostic model via an explainable machine learning pipeline and to stratify patients into molecular subtypes through consensus clustering. The central hub gene of this PA-associated signature was subsequently validated using spatially and temporally resolved single-cell data from the AD hippocampus. An AI-based drug-repurposing screen (Drugreflector) and molecular docking simulations were used to identify natural compounds targeting this hub gene for the treatment of AD. Finally, the dysregulation of this target was confirmed by quantifying its expression in peripheral blood samples from a clinical AD cohort.

**Results:**

We identified eight PA-associated gene signatures in AD patients that can help elucidate the pathogenesis and molecular stratification. *HDAC3* was identified as the central pathogenic hub, mainly distributed in astrocytes. Through drug screening, nervonic acid was identified as a potential therapeutic agent, showing a high binding affinity to HDAC3. Clinical validation confirmed elevated HDAC3 levels in AD patients.

**Conclusion:**

Our study reveals a novel PA-associated molecular axis in AD, with HDAC3 serving as a central epigenetic-metabolic regulator within astrocytes. This co-expression axis provides a framework for patient subtyping, offers a promising diagnostic biomarker, and identifies a potential therapeutic target for AD.

## 1 Introduction

Alzheimer’s disease (AD), the leading cause of dementia, imposes an escalating socioeconomic strain worldwide (Lane et al., 2018). Its pathological hallmarks, amyloid-beta plaques and tau neurofibrillary tangles, can trigger a cascade of synaptic failure, neuroinflammation, and neuronal loss (Twarowski and Herbet, 2023). Despite significant progress, the intricate metabolic underpinnings that influence disease onset and progression remain poorly defined, particularly regarding non-neuronal contributions.

Recent evidence has shifted the focus toward the critical roles of cellular metabolism and glial biology in AD. The pentose phosphate pathway (PPP), a metabolic shunt branching from glycolysis, is central to cellular homeostasis, as it generates NADPH for antioxidant defense and ribose-5-phosphate for nucleotide synthesis (Bar et al., 2025). PPP dysregulation is implicated in AD, contributing to oxidative stress and aberrant lipid metabolism (Palmer, 1999). Concurrently, astrocytes, the brain’s primary metabolic supporters, undergo functional alterations in AD, termed reactive astrocytosis, which compromises their ability to support synapses and buffer oxidative damage (Sadick et al., 2022). An emerging concept is that metabolic disruptions within astrocytes, particularly in glucose utilization pathways like the PPP, may dictate their functional state and influence neurodegeneration (Zhang et al., 2023). However, the specific molecular axis coupling pentose phosphate metabolism to astrocyte reactivity in AD has not been systematically explored.

In this study, we aimed to determine whether a shared signature representing PPP-astrocyte (PA) co-expression crosstalk exists and holds pathological significance in AD. We employed an artificial intelligence (AI)-powered multi-omics integration approach to identify this signature, characterize its diagnostic and stratification utility at the bulk level, and validate its cellular specificity and functional implications using single-cell transcriptomics and *in vitro* assays. Our ultimate goal was to establish a new molecular framework linking astrocyte metabolic reprogramming to AD pathogenesis.

## 2 Materials and methods

### 2.1 Source of bulk data

Transcriptomic datasets from post-mortem human hippocampal tissue were obtained from the GEO database. Dataset GSE48350 included 43 control and 19 AD samples (Wang et al., 2024). GSE28146 comprised 8 control and 22 AD samples (Wang et al., 2022). GSE29378 served as the training set, comprising 32 control and 31 AD samples, while GSE36980 (comprising 8 control and 10 AD samples) was designated as the independent validation set (Miao et al., 2025; Liu et al., 2024). Raw expression data were normalized using the limma R package in R (Ritchie et al., 2015). The PPP-associated gene list was obtained from the GeneCards database using a relevant score threshold greater than 1.

### 2.2 Limma analysis

Differential expression analysis between the AD and control groups in GSE48350 was conducted using the limma package in R (Ritchie et al., 2015). Genes with a log2FC >0.5 and a *p*-value < 0.05 were considered DEGs (Ritchie et al., 2015). The PPP-associated gene list was intersected with the DEGs to identify PPP-associated DEGs. Next, the PPP-associated DEGs were analyzed by KEGG and GO enrichment analysis using the clusterProfiler R package with reference to KEGG and GO gene sets downloaded from the MSIGDB database (Yu et al., 2012). Their expression pattern was depicted using the complex heatmap R package in R (Gu, 2022). In addition, we also performed a PPP-associated molecular interaction analysis using the STRING database and Cytoscape. xCell and WGCNA analysis.

The relative abundance of astrocytes in the GSE28146 hippocampal samples from patients with AD compared with HC was estimated using the xCell algorithm in R, which provides a score for the enrichment of various cell types based on gene expression profiles (Aran et al., 2017). To construct a gene co-expression network linked to astrocyte abundance, WGCNA was performed using the WGCNA R package on the GSE28146 dataset (Langfelder and Horvath, 2008). A soft-thresholding power was selected to ensure a scale-free topology (Langfelder and Horvath, 2008). Modules were identified via hierarchical clustering (minimum module size = 60). The module whose eigengene showed the strongest Pearson correlation with the astrocyte score was chosen as the astrocyte-associated module. The genes from this module were intersected with the PPP-associated DEGs to define the PA-associated shared DEGs.

### 2.3 Consensus clustering analysis

Based on the expression values of the PA-associated shared DEGs in AD samples, consensus clustering was performed using the ConsensusClusterPlus R package (Hu et al., 2022). The optimal number of clusters (k) was determined by examining the consensus matrix and the cumulative distribution function (CDF) curves (Hu et al., 2022). Differences in the expression of the PA-associated signature genes and immune infiltration profiles between the identified subtypes were compared via complex heatmap and the CIBERSORT package in R (Huang et al., 2024). GSEA was conducted using the clusterProfiler R package with reference to KEGG and GO gene sets downloaded from the MSIGDB database (Yu et al., 2012).

### 2.4 Explainable machine learning analysis

A machine learning workflow was implemented using the caret R package on the training set GSE48350 and validation set GSE29378 (Wu et al., 2024). The 132-model machine learning framework typically represents a comprehensive grid search approach that systematically combines multiple classification algorithms with various feature selection methods and preprocessing strategies (Wu et al., 2024). Combinations of these algorithms were applied to the MetaGSE dataset for signature selection and model construction using 10-fold cross-validation (Wu et al., 2024). The optimal model was selected based on the highest mean area by achieving a high AUC-index (Wu et al., 2024). Its performance was validated by ROC and PR analysis in the training and validation sets (Wu et al., 2024). A nomogram was built using the R rms package to examine the efficacy of model performance (Wu et al., 2024). SHAP analysis was performed to interpret the model and identify the most influential feature among the PA signature genes for defining the hub gene (Zhou et al., 2026).

### 2.5 Single-cell analysis

Single-cell RNAseq data from AD hippocampal tissues (GSE163577, sample = 9) were downloaded from the GEO database and processed using the Seurat R package (Huang et al., 2024). Quality control (QC) excluded cells with unusual features (<200 or >6,000) or high mitochondrial content (>15%) (Huang et al., 2024). Data normalization and identification of variable features were followed by PCA, t-SNE, and UMAP graph-based clustering (Huang et al., 2024). Cell types were annotated using the SingleR package in R with the Human Primary Cell Atlas (Zhang et al., 2022). The AUCell algorithm in R was used to score the activity of the PPP gene set across different cell types (Li C. et al., 2025; Li Y. et al., 2025). The expression of the PA hub gene was visualized on a dot plot. The *scTenifoldKnk* package was used to construct a gene regulatory network (GRN) from the single-cell RNA-seq data via tensor decomposition and simulate the removal of a target gene (Osorio et al., 2022). Briefly, a *k*-nearest neighbor graph was built using the UMAP embeddings of the target cell production (*n* = X cells) (Osorio et al., 2022). A GRN was inferred for all expressed genes using the tenfold function with default parameters (Osorio et al., 2022). The target gene was then virtually knocked out by setting its expression to zero, and the perturbed GRN was recomputed (Osorio et al., 2022). Differential network analysis identified genes whose regulatory connections changed significantly after knockout, defined by a normalized knockout effect score >0.5 (the default threshold) (Osorio et al., 2022). The top 10 most perturbed genes (ranked by effect score) were subjected to GO and KEGG pathway enrichment and GENEMINIA analysis using the clusterProfiler R package (FDR < 0.05) (Yu et al., 2012; Zhao et al., 2021). Pseudotime trajectory analysis of astrocytes was performed using the Monocle2 package in R (Fang et al., 2022).

### 2.6 Drug enrichment analysis

To systematically identify potential therapeutic compounds targeting AD, the DrugReflector platform in R and the TCMSP database were applied to the GSE48350 dataset (Ma, 2026). Each compound was scored using the Drug-Target Association Score (DTAS), which combines gene expression similarity, chemical structure similarity, known drug–target interactions, and network topological features (Ma, 2026). DTAS ranked all candidates in descending order, and the initial shortlist comprised compounds with a DTAS >0.9 and within the top 10 (Ma, 2026). For molecular docking, the 3D structure of the hub protein was obtained from the PDB database, and the structures of the candidate compounds were retrieved from the PubChem database (Wang et al., 2022). Docking simulations were executed using AutoDock Vina software, and the resulting binding poses were visualized using PyMOL software (Wang et al., 2022).

### 2.7 Clinical estimation

Peripheral blood specimens were collected in EDTA-coated tubes (Vazyme, China) from 12 participants, comprising 6 patients with AD and 6 HC. Peripheral blood mononuclear cells (PBMCs) were subsequently isolated by density gradient centrifugation. Briefly, each 2 mL blood sample was gently mixed with 10 mL of lymphocyte separation medium (Beyotime, China) in a 1:5 ratio. The mixture was incubated on ice for 15 min with intermittent gentle mixing, followed by centrifugation at 2100 × *g* for 10 min at 4 °C. After discarding the supernatant, the leukocyte-containing pellet was resuspended in 4 mL of fresh separation medium and centrifuged again under identical conditions (4 °C, 2100 × *g*, 10 min). The final PBMC pellet was obtained after removal of the supernatant. The study protocol was approved by the Institutional Ethics Committee, and all participants provided written informed consent was provided by all participants prior to sample collection. Total RNA was isolated from the PBMCs using TRIzol reagent (Takara, Japan). Reverse transcription to synthesize cDNA was performed using a PrimeScript RT Reagent Kit (Vazyme, China). Quantitative real-time PCR (qRT–PCR) analysis was then performed using TB Green Premix Ex Taq II (Vazyme, China) on a real-time PCR system. The sequences for all primers used are provided below:

HDAC3:
F-5′-CTCTCGGTGTCGGAATGTC-3′.
R-3′-AGGATCAGCAGGAACAGAGC-5.
GAPDH:
F-GCGACTTCAACAGCAACTCCC.
R-CACCCTGTTGCTGTAGCCGTA.

### 2.8 Statistical analysis

All statistical analyses were performed using R (version 4.2.2) and GraphPad Prism (version 9.0). Two-group comparisons were evaluated using an unpaired Student’s *t*-test, or the Mann–Whitney *U* test was applied. For comparisons among multiple groups, a one-way ANOVA followed by Tukey’s *post hoc* test was used. A *p*-value of < 0.05 was considered statistically significant.

## 3 Results

### 3.1 Identification of pentose phosphate pathway-associated DEGs for AD patients

**Figure 1:**
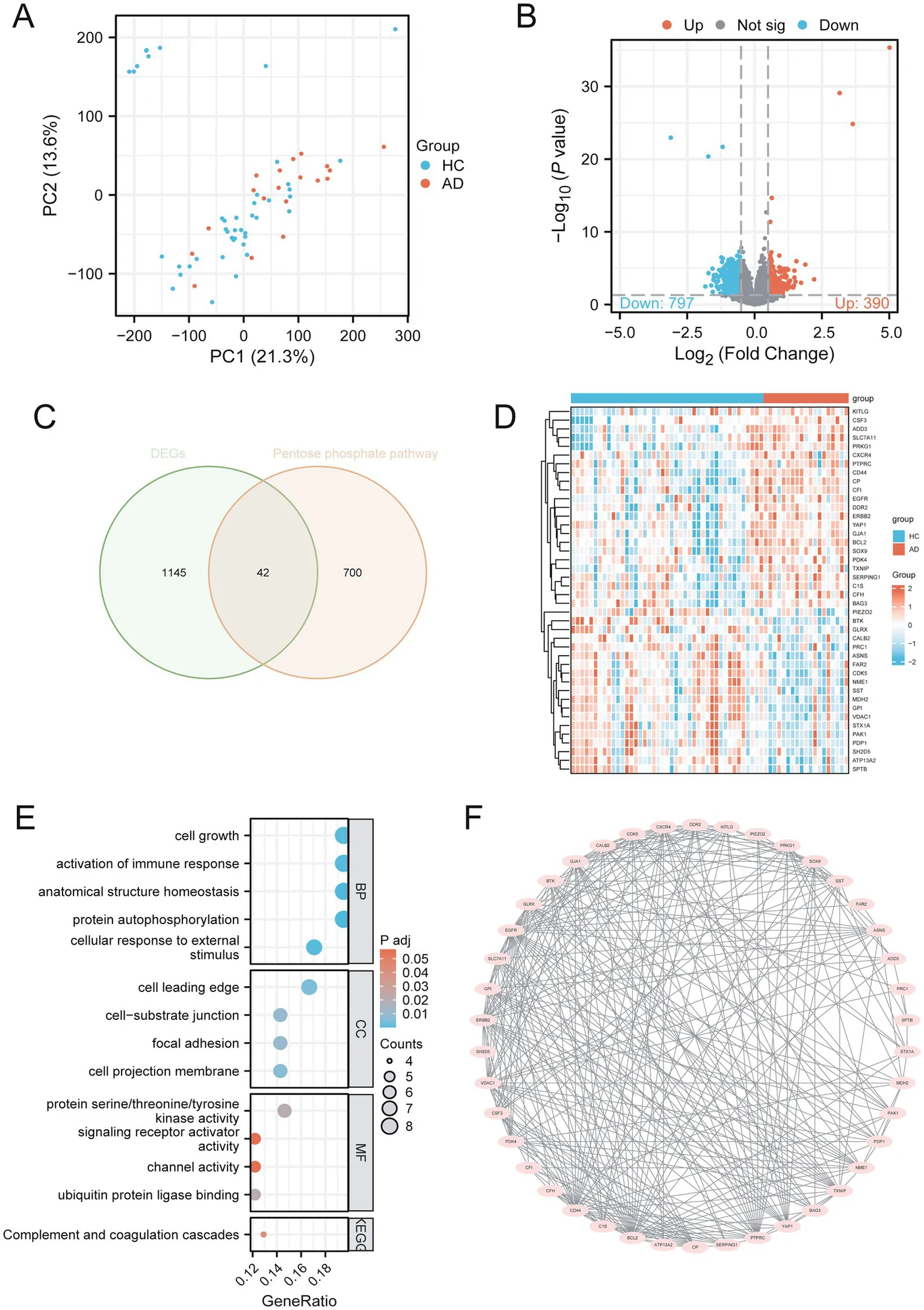
Identification of PPP-associated DEGs in AD. **(A)** PCA plot of the GSE48350 dataset. **(B)** Volcano plot detailing DEGs in GSE48350. **(C)** Venn diagram of the intersection between the DEGs and the PPP gene set. **(D)** Expression heatmap of PPP-associated DEGs. **(E)** KEGG and GO enrichment analyses of the PPP-associated DEGs. **(F)** PPP-associated DEGs interaction patterns.

After preprocessing GSE48350, differential analysis of the GSE48350 dataset identified 1,187 DEGs (Figures 1A–C). Intersection with the PPP gene set yielded 42 pentose phosphate pathway-associated DEGs (Figures 1C,F). Heatmap visualization revealed a clear divergence in the expression of these genes between AD and control hippocampi (Figures 1E,D), suggesting their relevance to AD metabolic dysregulation.

### 3.2 Identification of astrocyte-associated DEGs for AD patients

**Figure 2:**
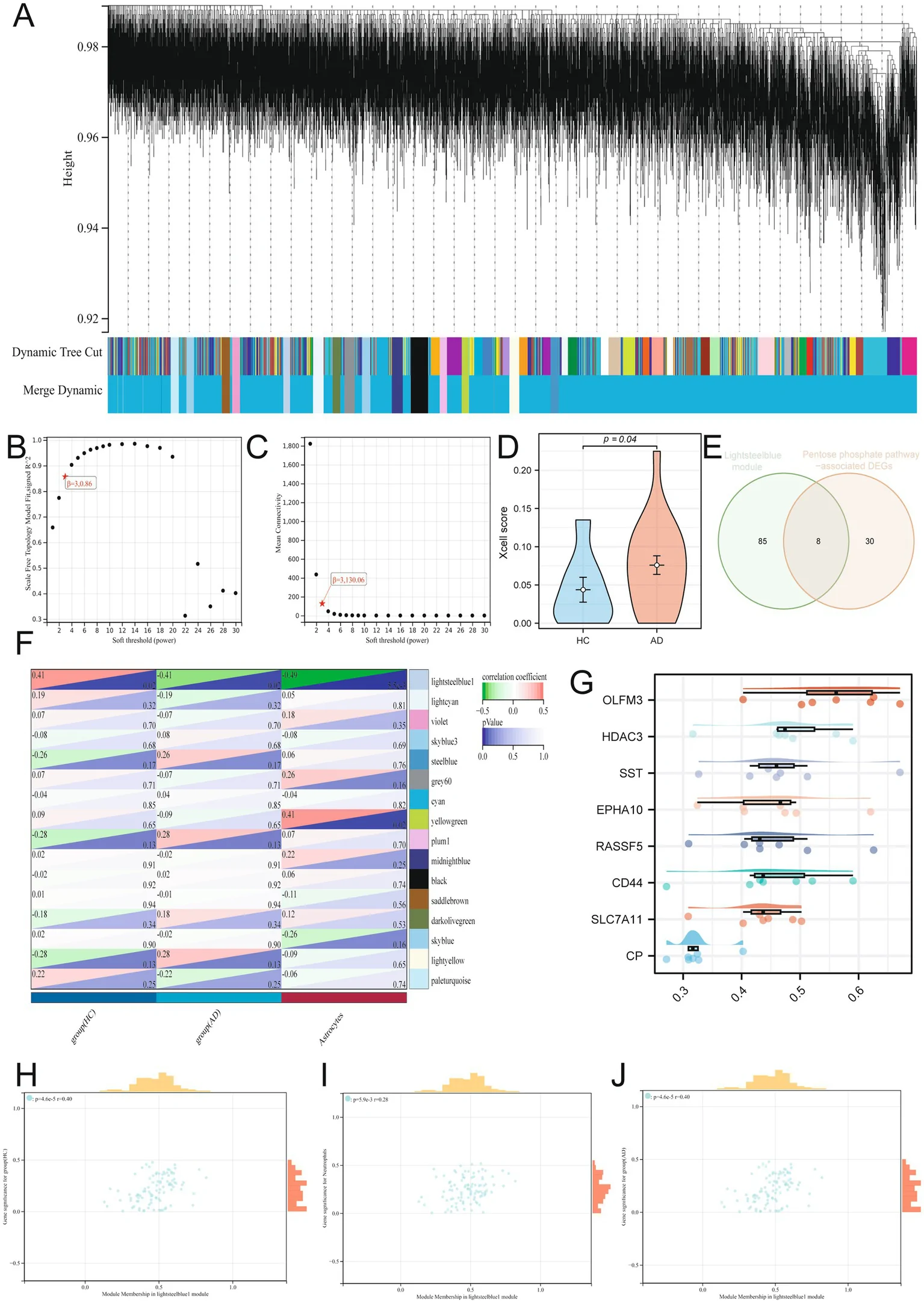
WGCNA identifies an astrocyte-associated module and defines the PA-associated signature. **(A)** Denogram cutting tree of WGCNA analysis. **(B–C)** *β* threshold of WGCNA analysis. **(D)** xCELL analysis of astrocyte infiltration. **(E)** Venn diagram illustrating the intersection leading to the eight-gene PA signature. **(F)** Correlation heatmap of WGCNA analysis. **(G)** Friend analysis of eight PA-associated DEGs. **(H–J)** Correlation dot plot of lightsteelblue1 module.

The xCell analysis of GSE28146 revealed a significantly higher astrocyte enrichment score in AD hippocampal tissue compared to controls (Figure 2D). WGCNA on GSE28146 identified 16 co-expression modules with *β* thresholds as 30.86 and 3130.6 (Figures 2A–C,F). The lightsteelblue1 module exhibited the strongest positive correlation with both astrocyte score and AD status (Figures 2H–J). This module contained 93 genes. The intersection of genes from this module with the 42 PPP genes yielded eight core PA-associated signature (Figures 2E,G). This signature comprises a subset of PPP genes modulated by astrocyte biology.

### 3.3 Identification of PA-related molecular subgroups for AD patients

**Figure 3:**
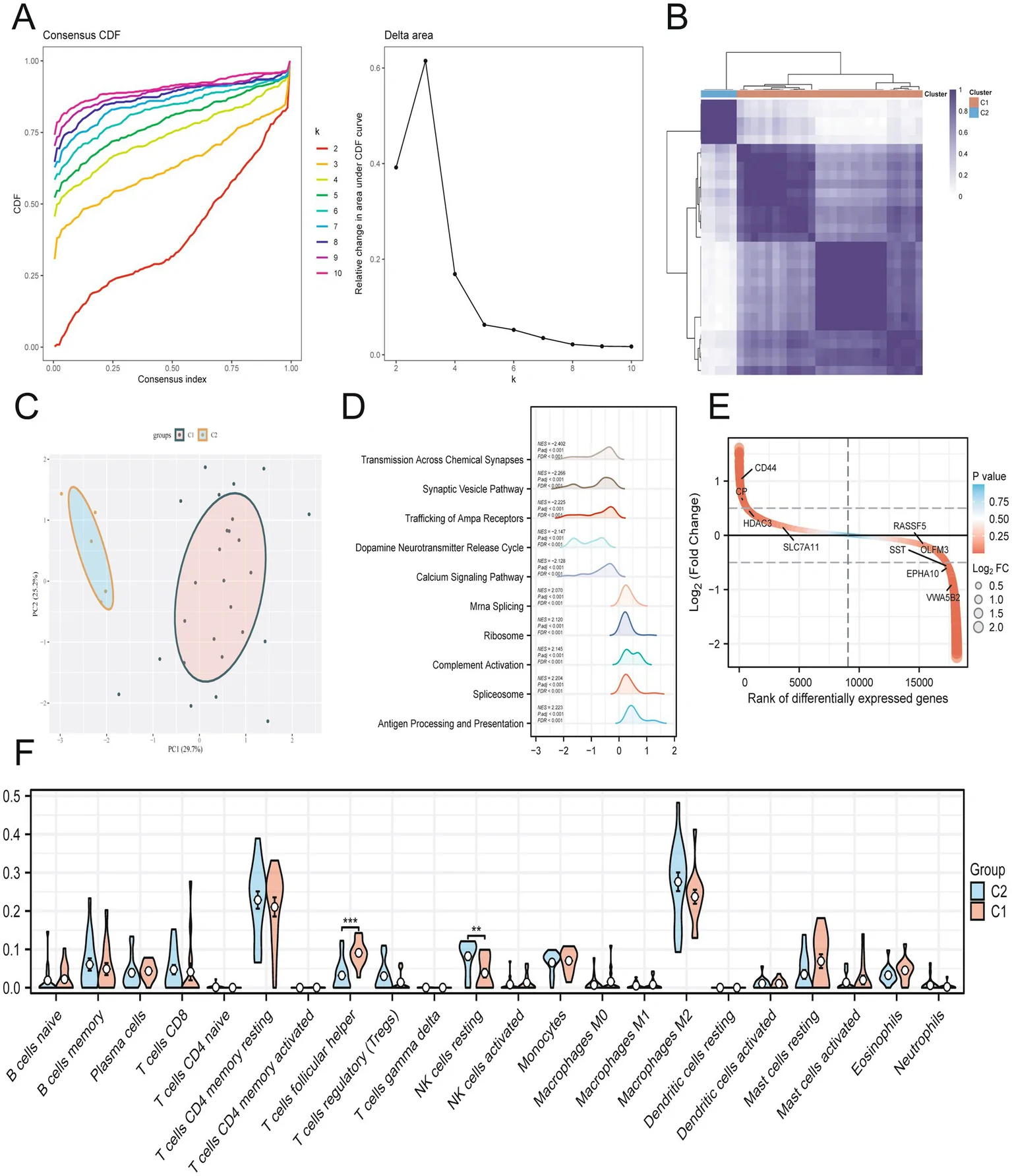
Molecular subtyping of AD patients based on the PA-associated signature. **(A–B)** Consensus matrix heatmap for *k* = 2. **(C)** PCA plot showing the separation of C1 and C2 subtypes. **(D)** GSEA plots showing representative enriched pathways in C1 and C2. **(E)** Heatmap comparing the expression of the 8 PA-associated signature genes between subtypes. **(F)** Box plot of immune cell infiltration differences between C1 and C2 subtypes.

Consensus clustering based on the PA-related signature in the GSE48350 training set robustly divided AD patients into two molecular subtypes: C1 and C2 (Figures 3A,B). PCA confirmed the clear separation of these subgroups (Figure 3C). The PA signature gene expression between C1 and C2 was assessed (Figure 3E). The GSEA analysis indicated that the C1 subtype was enriched for pathways related to epigenetic modification, whereas the C2 subtype was enriched for synaptic signaling and neurotransmitter pathways (Figure 3D). The CIBERSORT analysis revealed that the C1 subtype exhibited increased activation of specific immune cells, including T follicular helper cells and NK cells (Figure 3F).

### 3.4 Identification of a PA-related diagnostic model for AD patients

**Figure 4:**
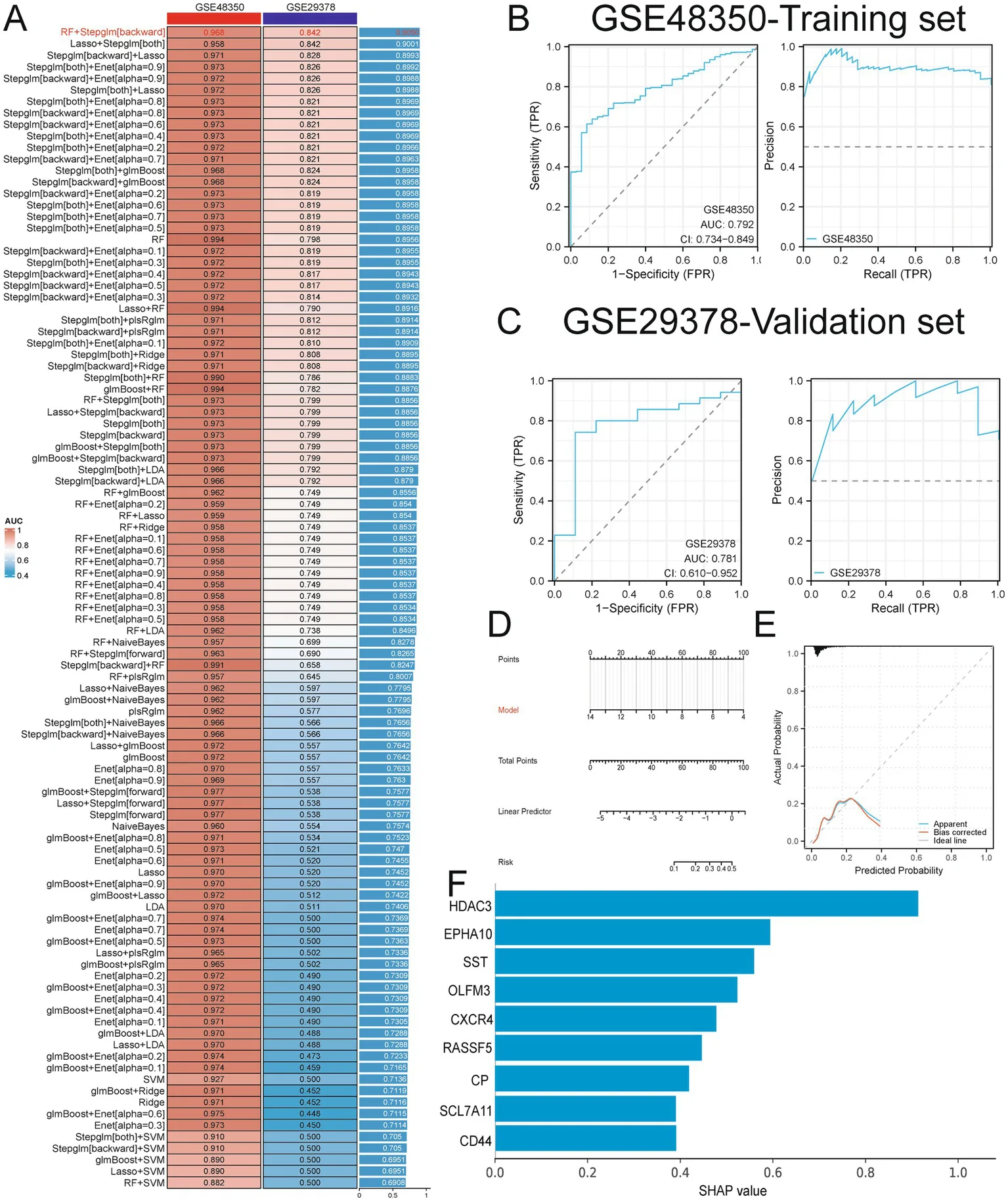
Development and validation of the PA-associated diagnostic model for AD patients. **(A)** Performance of 132 machine learning models. **(B)** ROC and PR curves on the GSE48350. **(C)** ROC and PR curves on the GSE29378. **(D)** Nomogram and calibration analysis of the model in GSE48350. **(E)** Calibration analysis of the model in GSE48350. **(F)** SHAP summary analysis in GSE48350.

A diagnostic model was constructed using a machine learning framework on GSE48350 and GSE29378. RF + Stepglm (backward) average AUC across 132 model combinations was high, with the selected model achieving an AUC of 0.9050 on the training set and AUC = 0.781 on the validation set (Figures 4A–C). Besides, a nomogram was created to provide a clinical tool based on the PA signature (Figures 4D–E). SHAP analysis clearly identified HDAC3 as the single most influential gene driving the model predictions, underscoring its role as the central PA hub gene (Figure 4F).

### 3.5 Single-cell atlas of astrocyte heterogeneity

**Figure 5:**
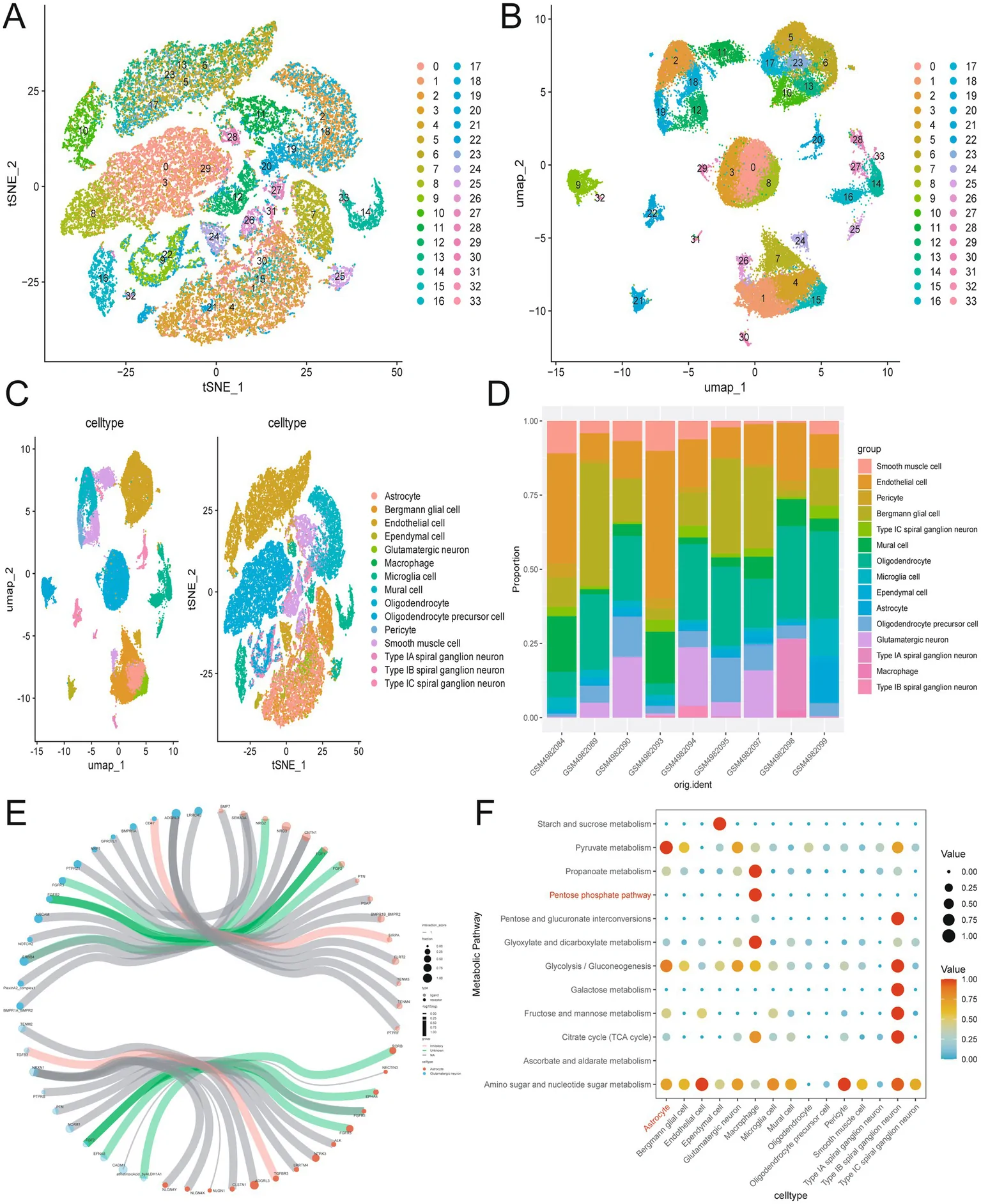
Single-cell characterization of the AD hippocampal microenvironment. **(A,B)** UMAP and t-SNE plots of cell clusters. **(C)** Cell type annotation of cell types. **(D)** Cell type proportions. **(E)** CellphoneDB analysis of astrocytes. **(F)** Metabolic heterogeneity among cell types.

Analysis of scRNA-seq data (GSE163577) resolved 33 cell clusters, which were annotated into 14 major cell types (Figures 5A–C). Astrocytes formed the relatively abundant cell population (Figure 5D). Focused analysis of the astrocyte cluster revealed a unique interaction between astrocyte and glutamatergic neuron, indicating distinct astrocyte states (Figure 5E). In addition, it can be witnessed that PPP was decreased in astrocytes (Figure 5F).

### 3.6 PA-related signature in astrocytes for AD patients

**Figure 6:**
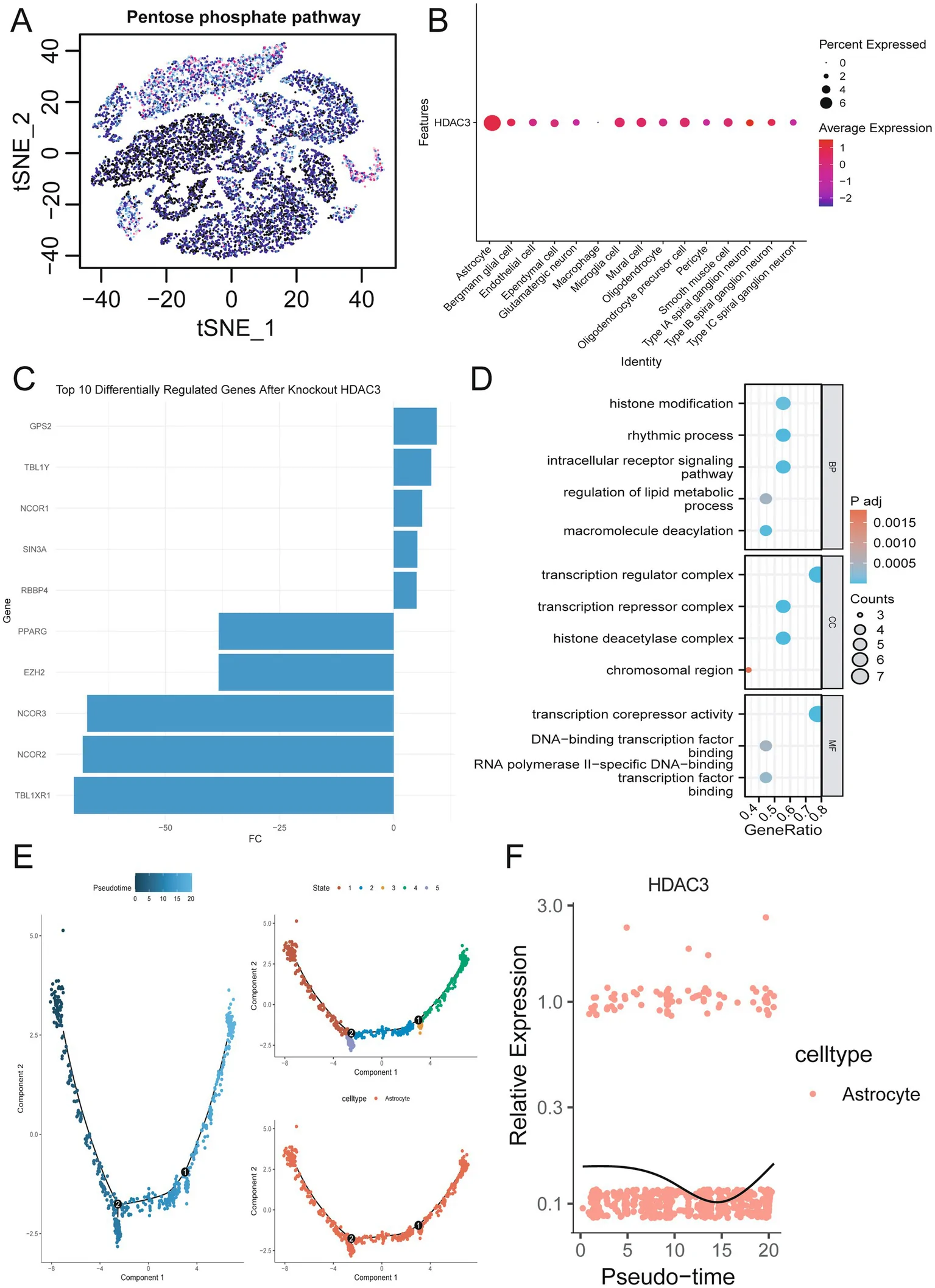
PA-associated signature and G6PD function in astrocytes, as inferred from scRNA-seq. **(A)** AUCell scores for the PPP. **(B)** Feature plot of HDAC3 expression among cell types. **(C–D)** Functional enrichment of genes perturbed by virtual HDAC3 knockout. **(E–F)** Pseudotime trajectory of astrocytes and HDAC3 in astrocytes.

AUCell scoring showed that PPP activity was significantly enriched in the astrocyte cluster (Figure 6A). Feature plot analysis confirmed that the expression of HDAC3 was predominantly concentrated in astrocytes (Figure 6B). Pseudotime analysis of astrocytes uncovered multiple differentiation trajectories, with HDAC3 expression gradually increasing along the path toward reactive astrocyte states (Figures 6E–F). *In silico* knockout of HDAC3 in astrocytes using the scTenfoldKnk tool perturbed a network of genes that were significantly enriched in functional pathways related to epigenetic modification (Figures 6C,D).

### 3.7 Therapeutic screening targeting PA-related signature and corresponding therapeutic agent enrichment

**Figure 7:**
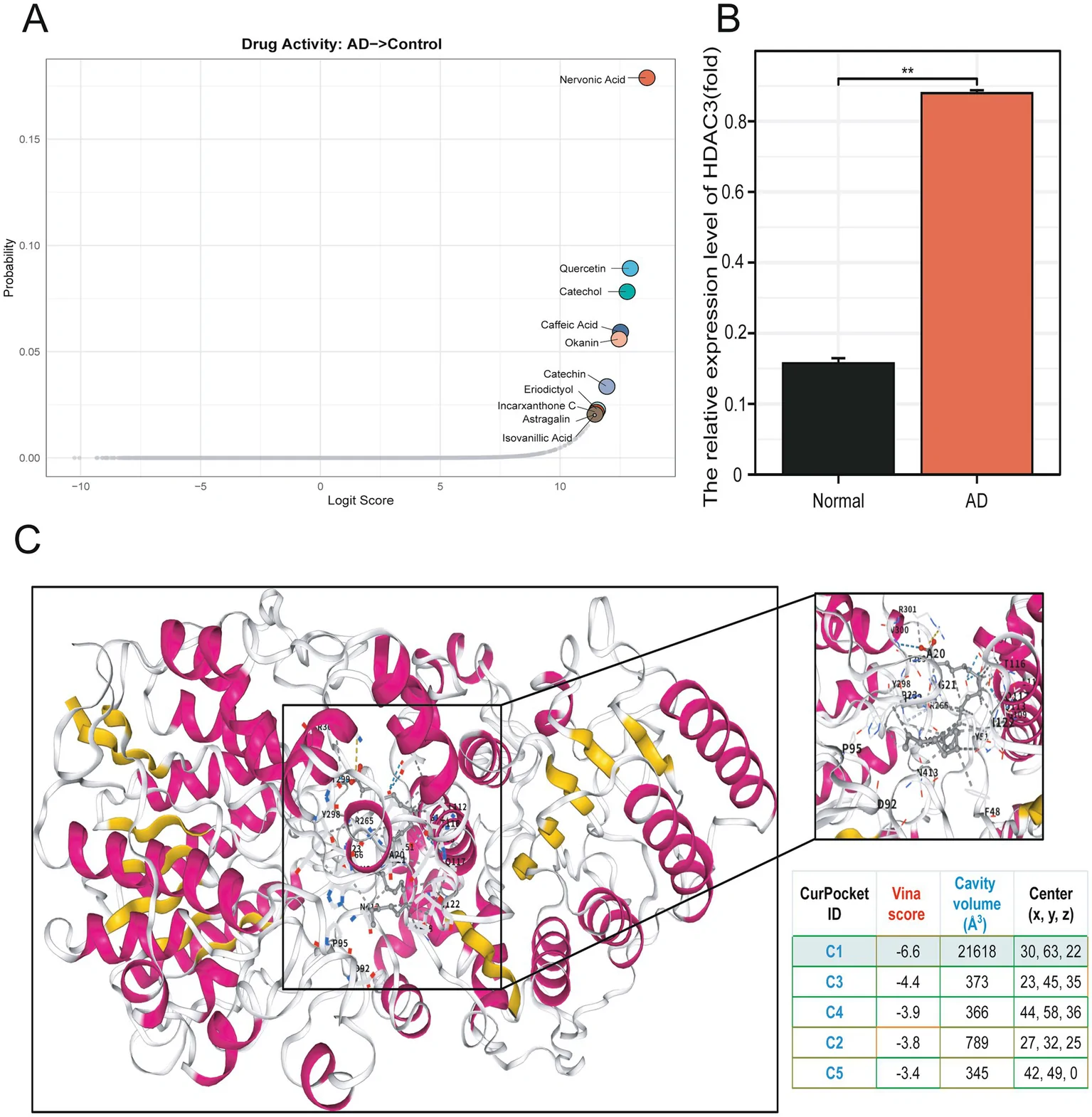
Therapeutic agent targeting AD and validation of HDAC3 expression. **(A)** Drug screening workflow and result. **(B)** qRT–PCR examination of HDAC3 expression in AD samples compared to HC. **(C)** Molecular docking analysis.

DrugReflector screening of the AD signature against the cMAP compound databases nominated nervonic acid as the top candidate, predicted to reverse the AD phenotype to that of an HC (Figure 7A). Molecular docking predicted a strong binding affinity between nervonic acid and the HDAC3 protein, with a binding energy of −6.6 kcal/mol (Figure 7C). To validate the functional role of HDAC3, we performed qRT–PCR analysis of clinical samples from AD patients compared to HCs, demonstrating an increased expression of HDAC3 (Figure 7B).

## 4 Discussion and conclusion

This integrative multi-omics study delineates a novel PA-associated axis in AD, identifying HDAC3 as its central epigenetic regulator. By integrating bulk tissue transcriptomics, single-cell mapping, and clinical validation, we have systematically established HDAC3 within astrocytes as a key node potentially linking metabolic pathway activity to disease classification and therapeutic intervention.

As a key class I histone deacetylase, HDAC3 compacts chromatin and represses gene transcription by deacetylating histone tails, serving as a core epigenetic regulator of cell fate and metabolic reprogramming (Li et al., 2023). In the context of AD, the upregulation of HDAC3, often reported in whole-brain or neuron-specific studies, has been associated with tau pathology, impaired synaptic plasticity, and cognitive deficits. Furthermore, clinical and animal model evidence suggests that its inhibition can ameliorate cognitive decline and neurodegenerative pathology (Li C. et al., 2025; Li Y. et al., 2025). In addition, existing evidence indicates that astrocyte reactivity involves extensive epigenetic remodeling (Clayton et al., 2024). For example, HDAC3 acts as a crucial epigenetic regulator, potentially modulating astrocytic inflammatory responses, metabolic adaptation, and neuro-supportive functions by repressing specific gene programs, with its activity confirmed to influence pro-inflammatory factor production in models of neuroinflammation (Zhang et al., 2016). While HDAC3-mediated regulation of metabolic pathways such as glycolysis is documented in cancer, its control over the PPP in the nervous system, particularly within astrocytes, remains poorly understood (Li et al., 2020). The PPP, a vital source of NADPH and ribose-5-phosphate, is tightly regulated at the transcriptional level (Shi et al., 2025). Upregulated HDAC3 may epigenetically repress the transcription of key PPP enzymes via histone deacetylation (Jung et al., 2025). This repression could lead to insufficient NADPH production, weakening cellular antioxidant defenses and exacerbating the inherent oxidative stress in the AD brain, while reduced ribose-5-phosphate might impair nucleotide synthesis and the biosynthetic capacity of astrocytes required under stress (Jung et al., 2025). Significantly, in our study, we performed computational knockout of HDAC3 in astrocytes of AD patients, indicating that the absence of HDAC3 may lead to the dysregulation of molecules and signaling related to the PPP. These results bridged the gap between HDAC metabolic regulation character without affecting protein modification.

In conclusion, our AI-driven multi-omics investigation successfully deciphered a PA-associated molecular signature in AD pathogenesis. We demonstrated that this signature is valuable for stratifying patients with AD into molecular subtypes with distinct pathological features and for constructing a robust diagnostic model. HDAC3 was unequivocally identified as the central hub of this axis, serving as a potential key epigenetic modulator in astrocytes. Furthermore, we nominated nervonic acid as a potential therapeutic agent targeting HDAC3, supported by computational docking and preliminary clinical validation of HDAC3 dysregulation. These findings position the PA-associated axis, and HDAC3 in particular, as a promising potential target for developing novel diagnostic strategies and epigenetic-metabolic therapies for AD. However, our study has several limitations. First, the diagnostic model and molecular subgroups of patients with AD identified in our study require validation in larger clinical cohorts to evaluate patient clinical characteristics and enhance the model’s reliability. In addition, although single-cell analysis provides multi-layer insights, it was based on a cohort of only nine patients with AD, which may not fully capture the heterogeneity of PPP in astrocyte states across the AD spectrum. Subsequent studies should incorporate higher-resolution single-cell and spatial profiling of patients with AD, combined with preclinical investigations that dynamically track PPP changes in astrocytes to elucidate AD pathogenesis. Moreover, the precise downstream molecular cascades of PPP in astrocytes that ultimately impair neuronal health through PPP regulation remain to be fully characterized in preclinical AD models. Future research should prioritize *in vivo* studies using conditional knockout or astrocyte-specific overexpression of HDAC3 in AD mouse models to elucidate the detailed mechanisms inferred from our *in silico* knockout analysis. Concurrently, high-throughput screening combined with AD patients’ clinical information for astrocyte-selective HDAC3 modulators could yield more targeted therapeutic candidates than existing pan-inhibitors. Similarly, the computationally identified therapeutic candidate warrants rigorous experimental validation in preclinical models to confirm its efficacy, optimal dosage, ability to cross the blood–brain barrier, and its capacity to modulate the proposed PA-associated axis and HDAC3.

## Data Availability

The datasets analyzed in this study are publicly available in the Gene Expression Omnibus (GEO) repository (https://www.ncbi.nlm.nih.gov/geo/). The accession numbers are as follows: GSE48350, GSE28146, GSE29378, GSE36980, and GSE163577. All data are freely accessible without restrictions. The custom analysis scripts generated during this study are available from the corresponding author upon reasonable request.

## Supplementary material

SUPPLEMENTARY FIGURE 1 Pre-processing of single-cell data. **(A−C)** QC metrics of single-cell data.
